# Regulation of Hematopoietic Stem Cell Activity by Inflammation

**DOI:** 10.3389/fimmu.2013.00204

**Published:** 2013-07-19

**Authors:** Laura G. Schuettpelz, Daniel C. Link

**Affiliations:** ^1^Department of Pediatrics, Washington University School of Medicine, St. Louis, MO, USA; ^2^Department of Medicine, Siteman Cancer Center, Washington University School of Medicine, St. Louis, MO, USA

**Keywords:** inflammation, hematopoietic stem cells, toll-like receptors, tumor necrosis factor, interferon

## Abstract

Hematopoietic stem cells (HSCs) are quiescent cells with self-renewal capacity and the ability to generate all mature blood cells. HSCs normally reside in specialized niches in the bone marrow that help maintain their quiescence and long-term repopulating activity. There is emerging evidence that certain cytokines induced during inflammation have significant effects on HSCs in the bone marrow. Type I and II interferons, tumor necrosis factor, and lipopolysaccharide (LPS) directly stimulate HSC proliferation and differentiation, thereby increasing the short-term output of mature effector leukocytes. However, chronic inflammatory cytokine signaling can lead to HSC exhaustion and may contribute the development of hematopoietic malignancies. Pro-inflammatory cytokines such as G-CSF can also indirectly affect HSCs by altering the bone marrow microenvironment, disrupting the stem cell niche, and leading to HSC mobilization into the blood. Herein, we review our current understanding of the effects of inflammatory mediators on HSCs, and we discuss the potential clinical implications of these findings with respect to bone marrow failure and leukemogenesis.

## Introduction

Infections and other inflammatory conditions place demands on hematopoiesis to increase production of immune effector cells. Hematopoietic cytokines and certain chemokines produced in response to infection are the primary mediators of the stress hematopoiesis response. They increase production of mature effects cells from lineage-committed hematopoietic progenitors and facilitate the mobilization of mature effector cells from the bone marrow to blood. Recent evidence suggests that hematopoietic stem cells (HSCs) also are direct targets of inflammatory signaling. In particular, interferons (IFNs), tumor necrosis factor (TNF), and toll-like receptor (TLR) ligands, among others, have been shown to stimulate the proliferation, differentiation, and repopulating ability of HSCs in multiple mouse models of infection and inflammation. Importantly, recent studies suggest that “inflammatory signaling” may also contribute to HSC regulation under homeostatic conditions (i.e., in the absence of overt infection or tissue damage). While inflammatory signaling in HSCs may be advantageous in the short-term, there is evidence that chronic inflammation may be deleterious to HSCs, and this may contribute to bone marrow failure and malignant transformation in humans.

Hematopoietic stem cells are a rare, quiescent population comprising only about 0.01% of bone marrow cells. These cells represent the foundation of the hematopoietic system, supplying the progenitors that give rise to all of the differentiated cell types in the blood. Although HSCs are largely quiescent, or dormant, at baseline (75% of long-term HSCs are in G_0_ phase of the cell cycle) (1), they can be induced to cycle and differentiate in response to various challenges to the hematopoietic system including chemotherapy, hemorrhage, and infection (2, 3). HSCs are defined by their ability to self-renew and support long-term (at least 12 weeks) multi-lineage hematopoietic engraftment. HSC-enriched populations can be identified by flow cytometry using a variety of cell surface markers and via exploitation of their staining properties with the vital dye Hoechst 33342 (4, 5). Kit^+^ lineage^−^ Sca^+^ (KSL) cells, while enriched for hematopoietic progenitor activity, only contain 5–10% HSCs. CD150^+^ CD48^−^ KSL and CD34^−^ KSL cells represent the two most commonly used murine HSCs phenotypes, each containing approximately 50% HSCs (5, 6). Of note, the study of HSCs in the context of inflammation or infection is complicated by the fact that the expression of the defining surface markers may be altered by inflammatory signals. For example, Sca-1 expression is induced by IFNs and TNF (7, 8), and c-Kit expression is markedly reduced in HSCs in response to the chemotherapeutic agent 5-fluorouracil (5-FU) (9). Thus, one must employ caution in interpreting the effects of inflammatory signals on HSCs, and functional studies are imperative to complement surface marker expression analyses.

**Figure 1:**
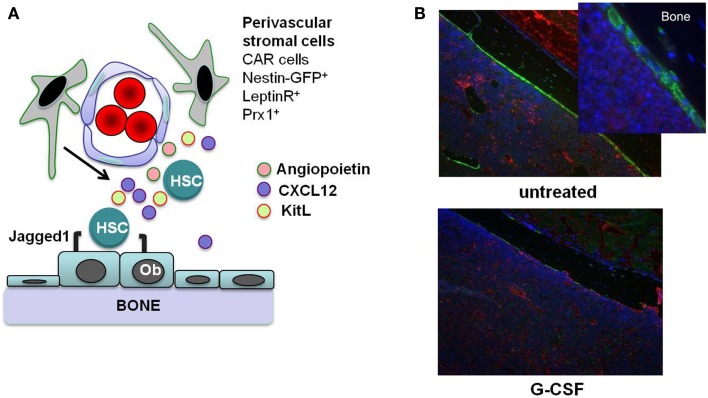
**Stem cell niches in the bone marrow**. **(A)** Hematopoietic stem cells (HSCs) reside in specialized niches in the bone marrow that are comprised of certain stromal cells, including endosteal osteoblasts (Ob) and perivascular stromal cells including CXCL12-abundant reticular (CAR) cells, leptin receptor^+^ cells, and nestin^+^ or Prx1-targeted mesenchymal progenitors. These cells maintain HSCs through the production of factors such as angiopoietin, Kit ligand (KitL), CXCL12, and Jagged1. **(B)** G-CSF treatment alters the HSC niches. Shown are sections from the femurs of Col2.3-GFP mice with osteoblast-specific GFP expression. After 7 days of G-CSF treatment (125 μg/kg given subcutaneously twice a day; bottom panel), there is a marked loss of osteoblasts (green color) compared to an untreated control animal (top panels). Original magnification 10×. Photo courtesy of Adam Greenbaum.

In this review, we focus on direct effects of inflammatory signals on HSCs. However, inflammatory signals also can alter the bone marrow microenvironment, which can indirectly affect HSCs. HSCs are localized to at least two anatomic regions in the bone marrow: the endosteum and perivascular region (5, 10–12). Stromal cells that populate these “stem cell niches” provide essential signals to HSCs that regulate their proliferation, differentiation, and retention in the bone marrow (Figure 1A). For example, Kit ligand (KitL) expression from endothelial cells and leptin receptor-positive perivascular stromal cells is required for HSC maintenance (13). Likewise, CXCL12 expression from mesenchymal progenitors and CXCL12-abundant reticular (CAR) cells is required for the efficient retention of HSCs in the bone marrow and maintenance of HSC repopulating activity and quiescence (14, 15). While HSCs largely reside within the bone marrow niches, they periodically traverse the bloodstream, and the number of blood-borne and extramedullary HSCs increases in response to inflammation or infection. Furthermore, multiple other tissues throughout the body are capable of supporting hematopoiesis, particularly under conditions of stress, inflammation, and infection (16). There is strong evidence that certain cytokines induced during inflammation indirectly affect HSCs through alteration of the bone marrow microenvironment. For example, granulocyte colony-stimulating factor (G-CSF) suppresses CXCL12 production from bone marrow stromal cells resulting in HSC mobilization into the blood (17–19). Thus, when considering the effect of inflammatory signals on HSCs, both inflammatory signaling in HSCs and alterations in the bone marrow microenvironment should be taken into account.

## Interferons

Interferons are cytokines produced by immune cells and others in response to pathogens (viruses, bacteria, parasites) and tumor cells. Type I interferons (IFN-α, IFN-β) are produced by a variety of cell types, including lymphocytes, dendritic cells, macrophages, fibroblasts, endothelial cells, and osteoblasts, and signal though the IFNα/β receptor (IFNAR) on target cells. Recently, Essers et al. (20) demonstrated that treatment of mice with IFN-α stimulated the *in vivo* proliferation of CD150^+^ CD48^−^ KSL cells. Both direct and indirect effects of IFN-α on HSC proliferation were observed. Importantly, while short-term (three doses) of IFN-α did not affect HSC repopulating activity in transplanted mice, chronic IFN-α stimulation (eight doses over 2 weeks) led to a decrease in CD150^+^ CD48^−^ KSL cells and a marked reduction in their repopulating activity. Consistent with these findings, Sato et al. (21) found that IFN-α induced HSC (KSL-side population cell) proliferation. Furthermore, they demonstrated that loss of interferon regulatory factor-2 (IRF2), a transcriptional repressor of IFN signaling, led to enhanced HSC cycling, and a reduction in repopulating ability in transplanted mice. Importantly, this repopulating activity was partially restored in *Irf2*^−/−^ HSCs if type I IFN signaling was disabled. Thus IRF2-mediated suppression of IFN signaling helps to maintain HSC quiescence and repopulating activity.

Like IFN-α, IFN-γ has been shown to regulate HSC proliferation and repopulating activity. Using a mouse model of *Mycobacterium avium* infection, Baldridge et al. (22) showed that this infection resulted in an IFN-γ-dependent increase in proliferation of HSCs (CD150^+^ KLS cells), and treatment of mice with IFN-γ alone was sufficient to induce HSC proliferation and mobilization. Furthermore, *M. avium* infection or IFN-γ treatment led to an HSC repopulating defect in transplanted mice. Interestingly, HSCs from IFN-γ-deficient (*Ifng*^−/−^) mice were more quiescent at baseline and had a repopulating advantage compared to wild-type HSCs. Similarly, infection with *Ehrlichia muris* results in an IFN-γ-dependent enhancement of HSC proliferation and a reduction in long-term repopulating activity (23).

Collectively, these data suggest that both IFN-α and IFN-γ directly stimulate HSC proliferation, and, if the exposure is prolonged, result in a loss of repopulating activity. IFN signaling also appears to play a negative role in regulating HSC quiescence and repopulating activity under basal conditions. Of note, there is evidence (at least for IFN-γ) that IFN signaling regulates HSC function in humans. Specifically, Yang and colleagues showed that treatment of CD34^+^ CD38^−^ human cord blood cells with IFN-γ markedly inhibits their ability to support multi-lineage hematopoiesis when transplanted into NOD-SCID mice (24).

## Tumor Necrosis Factor

TNF-α is a member of the TNF family of pro-inflammatory cytokines, produced largely by cells of the monocyte/macrophage lineage, but also by a variety of other cells including lymphocytes, natural killer cells, and endothelial cells. Originally identified as a serum-derived factor capable of inducing tumor cell necrosis (25), TNF-α is involved in a wide variety of other processes including stimulation of fever and regulation of cell proliferation and differentiation. TNF-α signals via two distinct receptors: the p55 receptor (*TNFRSF1A*), which is constitutively expressed in most tissues, and the p75 receptor (*TNFRSF1B*), whose expression is restricted largely to hematopoietic cells (26). There is general agreement that *ex vivo* treatment of murine and human hematopoietic progenitors with TNF-α inhibits their proliferation (27–30). For example, treatment of human CD34^+^ CD38^−^ cells with TNF-α *in vitro* suppresses hematopoietic colony formation (29) and the ability of these cells to sustain multi-lineage hematopoiesis after transplantation into NOD-SCID mice (27). The *in vivo* contribution of TNF signaling to HSC maintenance is more controversial. In the most recent and complete study, Pronk and colleagues carefully assessed HSC number and function in mice lacking *Tnfrsf1a*, *Tnfrsf1b*, or both (28). Although the number of phenotypic HSCs (KSL flk2^−^ cells) in the bone marrow is normal in all mice, transplantation experiments showed a modest increase in the long-term repopulating capability of either *Tnfrsf1a*^−/−^ or *Tnfrsf1b*^−/−^ HSCs, which was enhanced further using *Tnfrsf1a* and *Tnfrsf1b* doubly deficient cells. On the other hand, Rebel and colleagues reported that older mice (>6 months) lacking *Tnfrsf1a*^−/−^ had reduced repopulating activity compared to age-matched wild-type or *Tnfrsf1b*^−/−^ mice (31). To model the effect of increased TNF-α production during infection, Pronk et al. (28) assessed the effect of the *in vivo* administration of TNF-α on HSCs. They showed that short-term TNF (three doses) resulted in suppression of cycling HSCs and decreased HSC long-term repopulating activity. However, Rezzoug et al. showed that TNF-α production by bone marrow-derived CD8^+^ cells suppresses apoptosis of HSCs and facilitates hematopoietic engraftment after transplantation into allogeneic and syngeneic transplant recipients (32). Considering these somewhat discrepant results together, it is clear that the effects of TNF-α on HSCs are complex. It appears likely that the HSC response to TNF signaling is dependent on the dose and duration of TNF-α exposure and the local environment in which the HSCs reside, and there may be age-dependent differences in TNF response. That said, under basal conditions, it appears that TNF signaling negatively regulates HSC repopulating activity.

## Granulocyte Colony-Stimulating Factor

Granulocyte colony-stimulating factor is a cytokine produced by multiple hematopoietic and bone marrow stromal cell types in response to inflammatory signals, and it is the principle cytokine regulating neutrophil production. Systemic levels of G-CSF are increased in response to many types of infection (33), stimulating neutrophil production and release from the bone marrow. In addition to its prominent role in basal and stress granulopoiesis, G-CSF also regulates HSC function. G-CSF is a potent mobilizing agent, a property it shares with other inflammatory cytokines, including IL-6, IL-3, IL-12, and GM-CSF (34). There is a considerable body of literature showing that G-CSF induces the mobilization of hematopoietic stem/progenitor cells (HSPCs) primarily by altering the bone marrow microenvironment. G-CSF treatment results in marked changes in bone marrow stromal cells that have been implicated in HSC maintenance, including: (1) decreased CXCL12 expression from osteoblasts and Nestin-GFP^+^ stromal cells (35, 36); (2) decreased KitL and angiopoietin expression from Nestin-GFP^+^ stromal cells (36); and (3) osteoblast suppression (Figure 1B) (19, 35) The decrease of CXCL12 expression is of particular importance, since CXCL12 signaling regulates HSC quiescence, repopulating activity, and retention in the bone marrow (37–39). Indeed, recent studies show that conditional deletion of Cxcl12 from CAR cells (15) or leptin receptor^+^ stromal cells (14) in the bone marrow is sufficient to mobilize HSPCs into the blood.

Less well appreciated is the effect of G-CSF receptor signaling on HSCs. G-CSF receptor deficient (*Csf3r*^−/−^) mice at baseline have normal numbers of phenotypic HSCs in the blood but a marked long-term repopulating defect (40). Conversely, expression of a mutant G-CSF receptor with enhanced signaling properties confers a clonal advantage to HSCs upon G-CSF stimulation (41). G-CSF administration *in vivo*, despite mobilizing some HSCs to the blood and spleen, results in an absolute increase in phenotypic HSCs (CD150^+^ CD48^−^ KSL cells) in the bone marrow (42). Of note, this HSC expansion is not, however, associated with enhanced HSC activity, as the bone marrow of mice treated with G-CSF has significantly less repopulating activity than the bone marrow of untreated mice (43–45). Further study is needed to define the mechanisms by which G-CSF treatment inhibits HSC function.

In summary, G-CSF signals play an important role in maintaining HSC function under basal conditions. Increased G-CSF expression (either endogenous or pharmacologic) results in impaired HSC function in the bone marrow through alterations in the bone marrow microenvironment and possibly through direct G-CSF signaling in HSCs.

## Toll-Like Receptors

**Figure 2:**
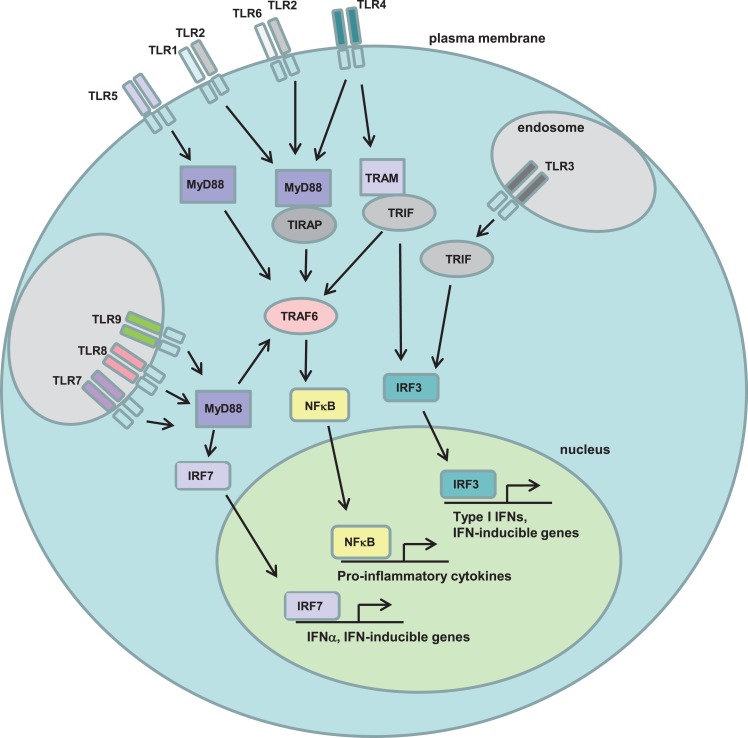
**Toll-like receptor signaling pathways**. Toll-like receptors (TLRs) are a family of transmembrane pattern recognition receptors that recognize a wide variety of pathogen- and danger-associated molecular patterns (PAMPs/DAMPs). TLRs are located either at the plasma membrane or in endosomes, and signal through either MyD88-dependent or TRIF-dependent pathways to active NFκB, IRF7, or IRF3 and induce the expression of pro-inflammatory cytokines.

Both mouse and human HSCs have been shown to express multiple members of the TLR family, a family of transmembrane pattern recognition receptors (PRRs) that detect pathogen-associated molecular patterns (PAMPs; e.g., lipopolysaccharide, single-stranded RNA, peptidoglycans). Twelve family members have been described in mice, and 10 in humans, and they play a central role in the innate (and subsequently the adaptive) response to pathogens such as viruses and bacteria. In addition, numerous non-pathogen associated ligands for TLRs have been described, the so-called danger-associated molecular patterns (DAMPs), which include intracellular molecules released upon necrotic cell death and extracellular matrix components that are either degraded or upregulated during tissue injury (46). All TLRs require intracellular adaptor proteins for signaling, with the adaptor MyD88 required for signaling through all TLRs except TLR3. TLR3 signaling requires the TRIF (aka TICAM1) adaptor, and TLR4 uses both a MyD88-dependent and a MyD88-independent (TRIF-dependent) pathway (Figure 2).

Nagai et al. (47) reported that murine bone marrow HSCs (Flk2^−^ KSL and IL7R^−^ KSL cells) express TLR2 and TLR4, and activation of HSCs *in vitro* with the TLR4 ligand LPS or the TLR2 ligand Pam3CSK4 led to MyD88-dependent myeloid differentiation and enhanced cell cycling. The same group later showed that chronic *in vivo* exposure to LPS altered phenotypic HSC populations and permanently impaired their repopulating and self-renewal capacities in transplantation experiments (48). In their study, low-dose LPS (6 μg daily injections) treatment for 4–6 weeks led to an expansion of the CD150^+^ CD48^−^ KSL and Flk2^−^ KSL HSC populations, as well as increased HSC cycling. When transplanted competitively into irradiated recipients, bone marrow from LPS-treated mice displayed impaired self-renewal and myeloid skewing compared to marrow from untreated mice. Finally, they noted that the changes observed with LPS treatment were reminiscent of HSC aging, including myeloid skewing and expansion of a CD150hi population lacking CD86 or CD18. Recently, Zhao et al. (49) similarly showed that chronic low-dose LPS (1 μg daily for 30 days) induced HSC cycling, increased HSC numbers and impaired their repopulating and self-renewal capacities in transplanted animals. In their study, LPS treatment was associated with increased transcription of Id1, encoding an inhibitory helix-loop-helix protein that was previously shown to be important for maintaining normal HSC numbers and repopulating activity (50), and loss of Id1 mitigated the LPS-induced HSC cycling and long-term repopulating defect. While these studies suggest that LPS increases HSC cycling while reducing repopulating activity, Rodriguez et al. (51) found that injection of mice with pseudomonas-derived LPS led to decreased cycling of KSL cells after 24 h. Also discordant, Takizawa et al. (52) reported that short-term treatment with higher-dose LPS (35 μg × 4 doses, each 2 days apart) led to increased HSC multi-lineage repopulating ability. Thus, the specific effects of LPS on HSCs may be dose and/or duration-dependent.

There also is evidence that TLR signaling may regulate HSC function in humans. Sioud et al. (53) demonstrated that, like murine HSCs, human bone marrow CD34^+^ cells express multiple TLRs, including TLR4, TLR7, TLR8, and TLR9. Furthermore, incubation of freshly isolated CD34^+^ cells with specific TLR ligands including immunostimulatory small interfering RNAs and the TLR7/8 ligand R848, led to the production of multiple cytokines (IL1-β, IL-6, IL8, TNF-α, GM-CSF), and induced the differentiation of CD34^+^ cells along the myeloid lineage in the absence of any exogenous cytokines. Likewise, human cord blood Lin^−^ CD34^+^ CD38^lo^ cells express multiple TLRs, including TLR1, TLR2, TLR3, TLR4, and TLR6 (54), and culture of these cells with the TLR1/2 agonist Pam3CSK4 stimulated proliferation and myeloid differentiation.

Taken together, these studies in mice and humans clearly demonstrate that HSCs express TLRs, and TLR ligation influences HSC cycling and promotes differentiation toward a myeloid fate. Further studies are needed to elucidate the effects of specific TLR ligands on HSCs, as well as determine the dose- and duration-related effects of TLR ligation on HSC proliferation and function. As with inflammatory cytokines, TLR signaling may alter the expression of HSC-defining surface markers. For example, CD150 is upregulated on multiple hematopoietic cell types in response to TLR signaling (55–57), and therefore the “expansion” of HSCs in response to TLR ligation may reflect an alteration in HSC-related surface marker expression of cells that are not true HSCs. Interestingly, bone marrow from *TLR4*^−/−^, *TLR9*^−/−^, and *MyD88*^−/−^ mice has a repopulating advantage when transplanted competitively with wild-type marrow into lethally irradiated recipients (58), suggesting that TLR signaling may contribute to the maintenance of HSCs under homeostatic conditions. This data suggests that endogenous TLR ligands, such as those produced by normal gut flora, may contribute to the regulation of baseline HSC activity. Further studies are necessary to explore this possibility and define the source of endogenous TLR ligands affecting HSCs in the absence of overt infection.

It is presently unclear whether the *in vivo* effects of TLR agonists on HSCs are direct, involving TLR signaling on the HSCs themselves, or indirect, requiring TLR signaling by another hematopoietic or stromal cell type. To address this issue, Megias and colleagues transplanted purified wild-type KSL IL7Rα^−^ cells into *TLR2*^−/−^, *TLR4*^−/−^, or *MyD88*^−/−^ mice and then injected these recipients with specific TLR2, TLR4, or TLR9 agonists (59). They observed that TLR stimulation rapidly induced differentiation of transplanted KSL IL7Rα^−^ cells into macrophages. This approach removes the potential contribution of soluble mediators secreted by recipient cells, supporting the idea that HSCs may be directly influenced by TLR ligation. On the other hand, LPS-induced increased expression of ID1, which has been implicated in LPS-induced loss of HSC repopulating activity, is not mediated by direct TLR signaling in HSCs (49). Moreover, Shi et al. showed that treatment of mice with LPS led to increased expression of CCL2 by nestin-GFP^+^ stromal cells and CAR cells, two bone marrow stromal cell populations implicated in HSC maintenance (60). Thus, it is likely that TLR agonists regulate HSCs in both cell autonomous and non-cell autonomous fashions.

The regulation of TLR expression on HSCs is also not well understood. Of note, Joo et al. (61) reported that G-CSF mobilized HSCs have increased TLR2 levels compared to unmobilized bone marrow HSCs, and *ex vivo* treatment of Lin^−^ c-Kit^+^ bone marrow cells with G-CSF led to upregulation of TLR2 expression as detected by flow cytometry. Thus inflammatory cytokines produced during infection or tissue damage may help regulate TLR expression on HSCs, thus priming them to directly respond to the offending pathogen or damage-associated ligand. Additional studies are necessary to understand how G-CSF regulates TLR expression, and determine what other factors regulate TLR signaling in HSCs.

In addition to TLRs, several other classes of PRRs are important for direct pathogen and damage-associated pattern recognition including the C-type lectin receptors (CLRs), the nucleotide-binding oligomerization domain (NOD)-like receptors (NLRs), retinoic acid-inducible gene (RIG)-I-like receptors (RLRs), and purinergic receptors. Human CD34^+^ cells express the NLR NOD2, an intracellular PRR with a role in recognizing bacterial peptidoglycans and activating NF-κB. Stimulation of these cells with a NOD2 agonist led to increased expression of the PU.1 transcription factor, important for myeloid differentiation, and enhanced their responsiveness to TLR2 ligation with the production of multiple inflammatory cytokines (TNF-α, IL-1β, GM-CSF) (62). In addition, both mouse and human HSCs have been shown to express purinergic receptors, which respond to extracellular nucleotides released during tissue injury or inflammation. Exposure to the purinergic receptor ligands ATP or UTP enhanced the proliferation of human CD34^+^ HSCs (63), and inhibition of purinergic signaling mitigated the enhanced HSC cycling in a mouse model of inflammatory bowel disease (64).

## Inflammation and HSC Dysfunction in Human Disease

As discussed above, studies in mice clearly show that inflammatory cytokines and pathogen- or danger-associated ligands can influence the cycling status, differentiation, and repopulating activity of HSCs. While fewer studies have been performed using human cells, human HSCs clearly do, like murine HSCs, respond to similar inflammatory stimuli, and a link between inflammation and bone marrow dysfunction has long been observed. For example, increased expression of TNF-α and IFN-γ has been observed in the bone marrow of patients with MDS (65). Similarly, IFN-γ and TNF-α expression is higher in the bone marrow of patients with aplastic anemia compared to healthy controls (66). In a study of children with idiopathic aplastic anemia, bone marrow CD4^+^, and CD8^+^ cells expressing IFN-γ and TNF-α were significantly increased compared to normal controls, and a higher percentage of marrow TNF-α-expressing T cells correlated with an unfavorable outcome (67). Similarly, increased bone marrow levels of these cytokines are associated with Fanconi Anemia (FA), and inhibition of TNF-α restores erythropoiesis in a mouse model of FA (68). Li et al. (69) provided further evidence for a role for TNF-α in the pathogenesis of FA by demonstrating that, while TNF-α initially inhibited the growth of HSCs from FA mice (*Fancc*^−/−^), longer-term exposure promoted the generation of cytogenetically abnormal clones that led to acute myelogenous leukemia upon transplantation into congenic wild-type recipients.

Augmented TLR signaling has also been implicated in myelodysplastic syndrome (MDS) and acute myeloid leukemia (AML). TLR4 expression is increased in CD34^+^ cells from patients with MDS compared to healthy controls, and is associated with enhanced apoptosis (70). Similarly, the mRNA expression of TRAF6, a mediator of MyD88-dependent TLR signaling, is increased more than 10-fold in patients with MDS compared to healthy controls (71). Starczynowski et al. (72) demonstrated that upregulation of TLR signaling via loss of miR-145 and miR-146a contributes to myelodysplasia in 5q^−^ syndrome. In their study, they identified the TLR signaling pathway mediators TIRAP and TRAF6 as respective targets of these non-coding RNAs, and showed that knockdown of miR-145 and miR-146a together or enforced expression of TRAF6 in murine HSCs led to an MDS-like phenotype consisting of thrombocytosis, neutropenia, and megakaryocytic dysplasia. Furthermore, approximately one-third of mice transplanted with TRAF6-overexpressing HSCs ultimately developed bone marrow failure or AML.

Gain-of-function mutations of *MYD88* are common in certain lymphoproliferative syndromes. Ngo et al. (73) identified *L265P MYD88* mutations in 29% of 382 primary activated B-cell-like diffuse large B-cell lymphoma samples. They confirmed that this is a gain-of-function mutation, enhancing NF-κB, and JAK-STAT3 signaling and tumor cell survival. The same *MYD88* mutation was identified by a second group in 9 of 310 CLL patients (2.9%), and was associated with a younger age and more advanced clinical stage at diagnosis (74). Again, the mutation was associated with activation of downstream signaling effectors and enhanced cytokine secretion of tumor cells upon stimulation with TLR ligands. CLL cells have previously been shown to express multiple TLRs, and stimulation of these cells with TLR ligands protects them from apoptosis (75). More recently, *L265P MYD88* mutations were identified in>90% of patients with Waldenstrom macroglobulinemia and approximately 50% of patients with immunoglobulin M (IgM) monoclonal gammopathy of unknown significance (MGUS) (76, 77).

Collectively, these studies demonstrate a role for inflammatory cytokines and activated TLR signaling in the pathogenesis of human bone marrow diseases. Both normal and malignant HSCs are affected by these signals, though further studies are needed to further define the precise roles of individual inflammatory signals on HSCs in both normal and disease states. Furthermore, the finding of enhanced inflammatory signaling in these bone marrow disorders suggests that targeted interruption of various inflammatory pathways may provide therapeutic benefit. Indeed, immune suppression is a cornerstone of therapy for idiopathic bone marrow failure, although the agents used are widely suppressive and the precise mechanism of marrow failure is not well understood. Anti-TNF-α agents are widely used to treat inflammatory disorders such as rheumatoid arthritis (RA). While hematologic complications are relatively uncommon, there are reports of neutropenia and other forms of bone marrow suppression in patients receiving this type of therapy (78). Notably, Papadaki and colleagues reported an increase in bone marrow erythroid precursors in patients receiving anti-TNF-α therapy for RA, and thus, as with mice, the effects of this cytokine on human stem and progenitor cells may be complex (79). In contrast to anti-inflammatory therapies, pro-inflammatory cytokines are used therapeutically in the treatment of various infections and immune disorders (e.g., IFN-α in hepatitis and IFN-γ in chronic granulomatous disease), and yet the known effects of these agents on the survival, cycling status, differentiation, and repopulating ability of HSCs in mouse studies suggest that the long-term effects of such therapies on the bone marrow warrants further study. Of note, bone marrow suppression is a common side effect of interferon therapy, often requiring dose-reduction or the use of hematopoietic growth factors to maintain acceptable neutrophil, red blood cell and platelet numbers (80).

## Summary and Future Directions

**Figure 3:**
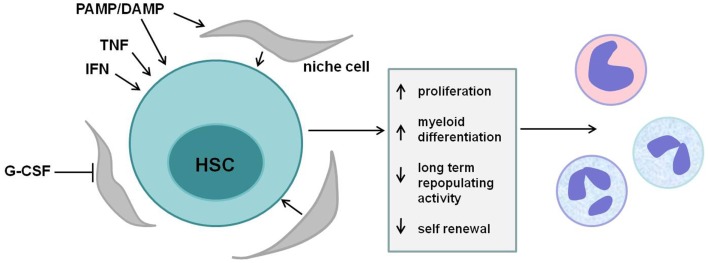
**HSC regulation by inflammatory mediators**. Multiple cytokines and pathogen-associated ligands regulate hematopoietic stem cells (HSCs). Tumor necrosis factor (TNF), interferons (IFNs), mobilizing cytokines such as granulocyte colony-stimulating factor (G-CSF), and various pathogen- and danger-associated molecular patterns (PAMPs/DAMPs) act either directly via their cognate receptors on HSCs or indirectly via stromal cells in the stem cell niche to affect HSCs. Short-term signaling induces HSC proliferation and myeloid differentiation, supplying effector cells of the innate immune response. Sustained exposure to these signals, however, reduces HSC long-term repopulating activity and self-renewal and may contribute to bone marrow failure and/or malignancy.

Accumulating evidence support a role for HSCs as truly “front line” players in the immune response. Pro-inflammatory cytokines and pathogen- or damage-associated molecules influence HSCs directly, shaping their proliferation status, lineage-bias, and repopulating ability (Figure 3). The acute response of HSCs to these signals is to stimulate the proliferation and production of myeloid cells, likely as a means to increase short-term production of innate immune cells. However, these inflammatory signals also lead to a loss of HSC self-renewal and repopulating capacity, and chronic inflammatory signaling in HSCs may contribute to bone marrow failure and/or hematopoietic malignancies.

While the evidence discussed above clearly suggest an active role for HSCs in the response to inflammatory signals, several important questions remain. In particular, the role of inflammatory cytokines and TLR signaling under baseline conditions is not clear. The finding of increased HSC quiescence in *Ifng*^−/−^ mice and a repopulating advantage of HSCs deficient for IFN-γ, TNF-α, or TLR signaling under normal conditions suggest that inflammatory signals may play a role in regulating the size of the HSC pool and the proliferation and repopulating potential of HSCs at baseline. Further studies are needed to elucidate the source of the endogenous ligands of these inflammatory signals and further delineate their role in maintaining HSCs. Commensal flora, for example, could conceivably provide a stimulus for TLR signaling. Clarke et al. (81) demonstrated that bacterial peptidoglycan could be found in the serum and bone marrow following gut colonization of otherwise germ-free mice, supporting the idea that normal host flora can provide systemic signals to immune cells. It is also worth considering that irradiation, the most common conditioning regimen used in mice, induces local expression of DAMPs and pro-inflammatory cytokines in the bone marrow (82). It is presently unclear what role these inflammatory mediators play in regulating engraftment, and whether the repopulating advantage seen with HSCs from mice lacking inflammatory signaling pathway components is influenced by the conditioning regimen of the recipient mice.

The effects of inflammatory signals on HSCs at baseline or during times of stress or infection are likely dependent upon the level and duration of signaling, with short-term exposures facilitating the development of an effective immune response and chronic signaling potentially contributing to HSC dysfunction. Further studies are necessary to determine these dose- and duration-dependent effects, as well as the effects of combinations of inflammatory mediators as would be present in most cases of infection or tissue injury. A more clear understanding of the effects of inflammatory signals on HSCs, both direct and indirect, as well as an understanding of the signals that are dysregulated in various human bone marrow diseases, will potentially provide an avenue for targeted therapies in these diseases by interfering with (or augmenting) such signals.

## Conflict of Interest Statement

The authors declare that the research was conducted in the absence of any commercial or financial relationships that could be construed as a potential conflict of interest.
